# Supplementary material for: Transient evolution of permeability and friction in a slowly slipping fault activated by fluid pressurization
Source: Nat Commun. 2022 Jun 1;13:3039. doi: 10.1038/s41467-022-30798-3 (PMC9160226; doi:10.1038/s41467-022-30798-3)
Supplement: Supplementary file 1 — Supplementary Information [file 41467_2022_30798_MOESM1_ESM.pdf]

Supporting Information for

Transient Evolution of Permeability and Friction in a Slowly Slipping Fault Activated by Fluid Pressurization

by Cappa et al.

Supplementary Figures 1 to 8

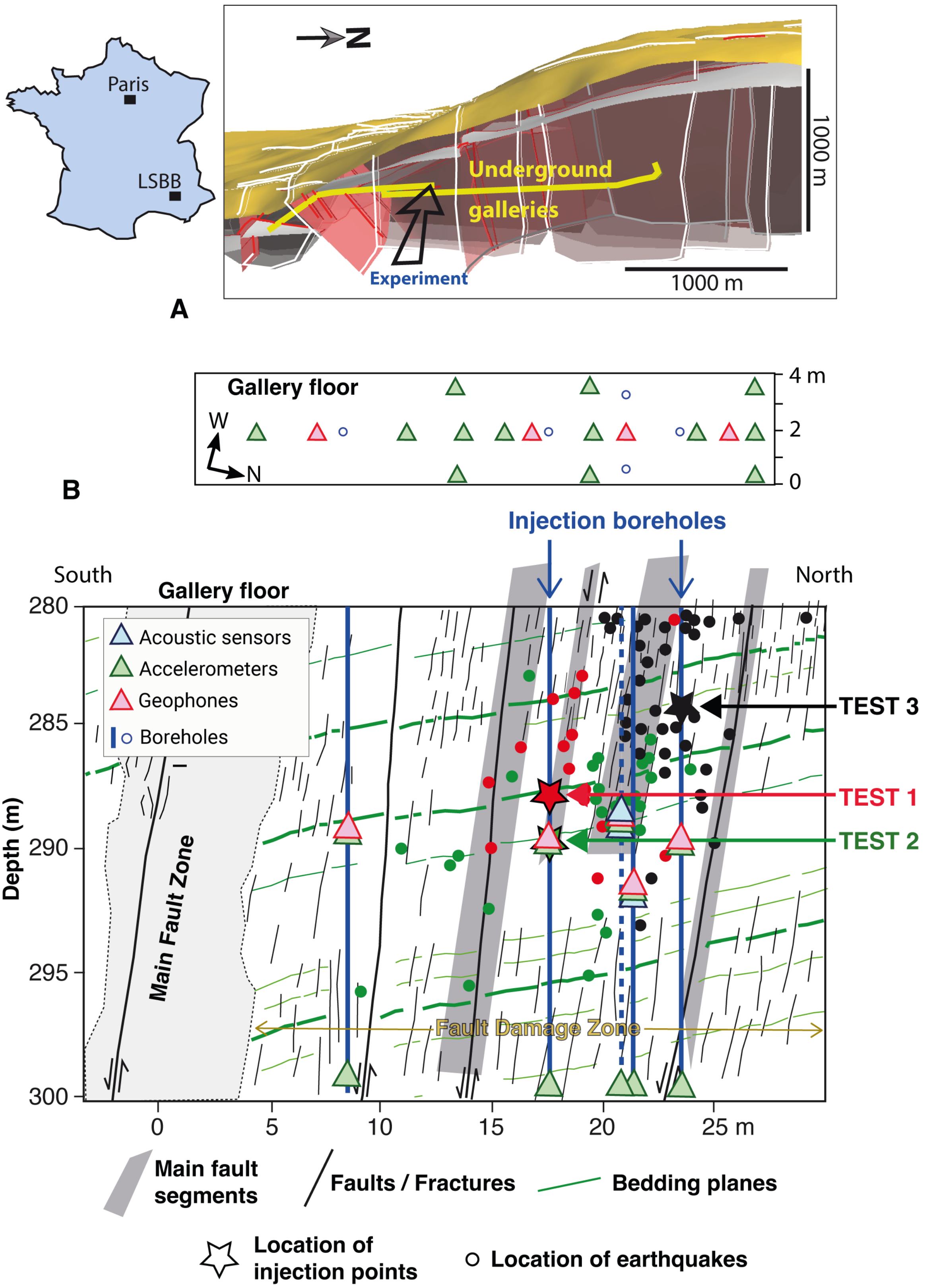

Supplementary Figure 1. **a** 3D view of the galleries (yellow) and the location of the experiment within the Low Noise Underground Laboratory in France. **b** Locations of seismic sensors on and below the gallery floor.

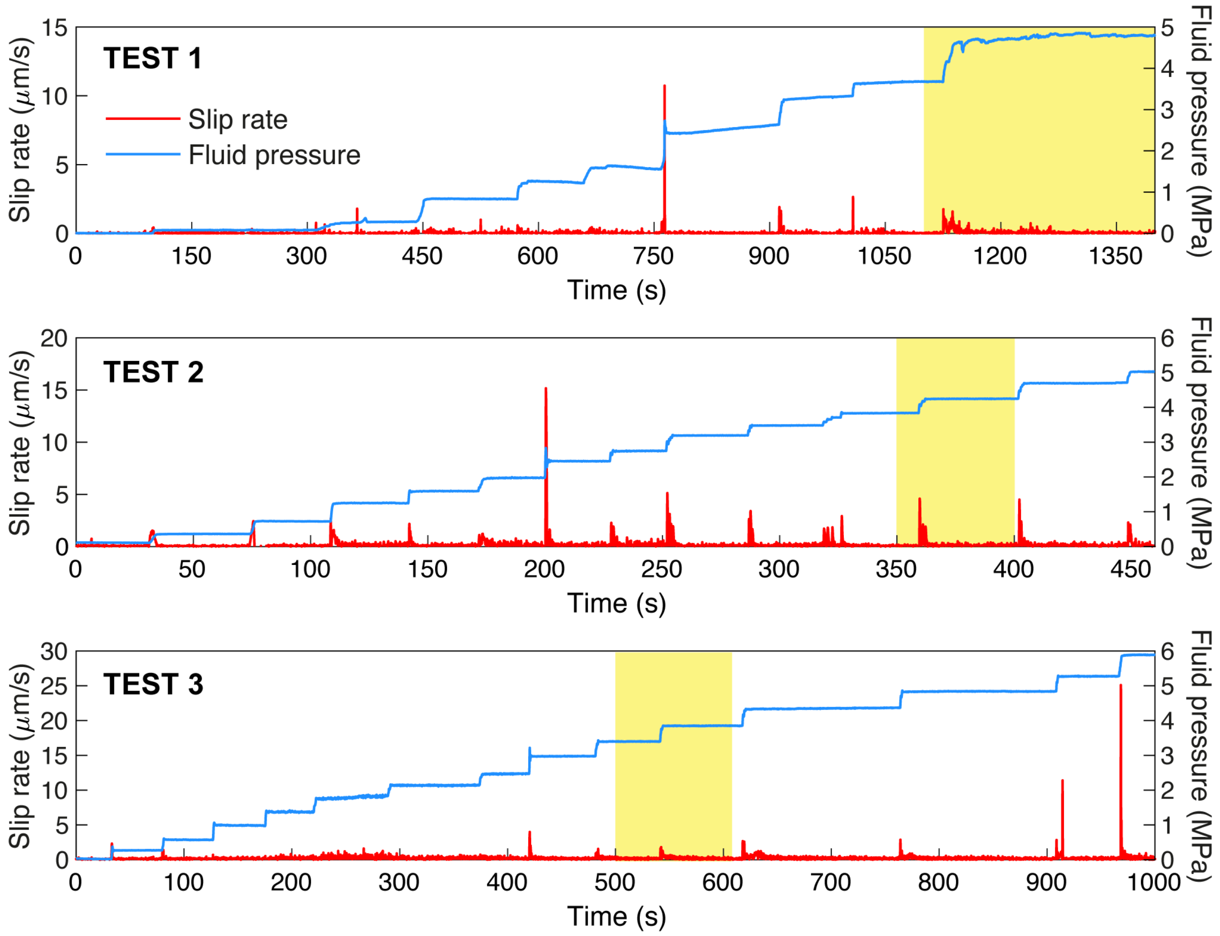

Supplementary Figure 2. Slip rate (red) and fluid pressure (blue) measured as a function of time for the three fluid injection experiments. The yellow boxes indicate the time window selected for the modeling.

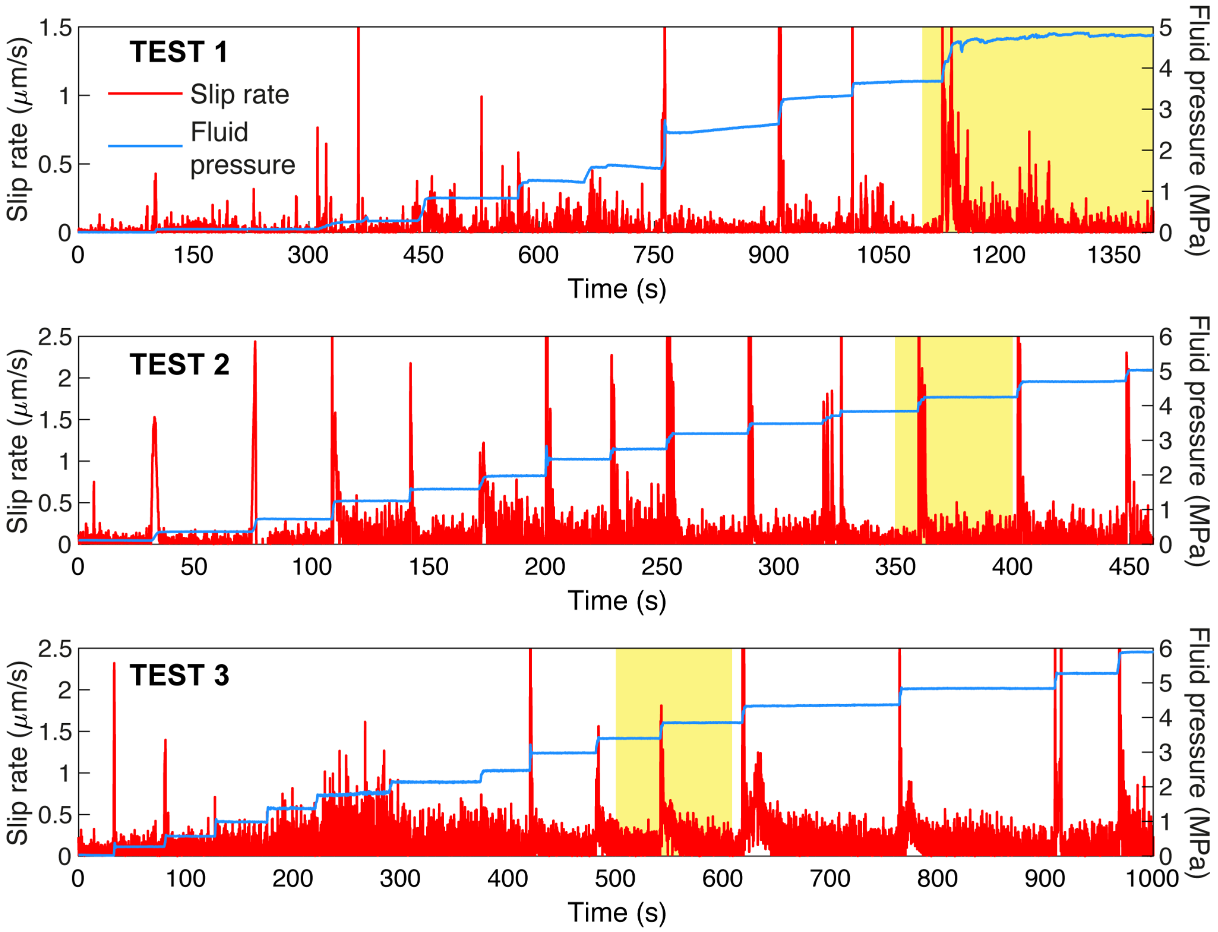

Supplementary Figure 3. Close-up view of slip rate (red) and fluid pressure (blue) as a function of time for the three fluid injection experiments shown in Figure S2. The yellow boxes indicate the time window selected for the modeling.

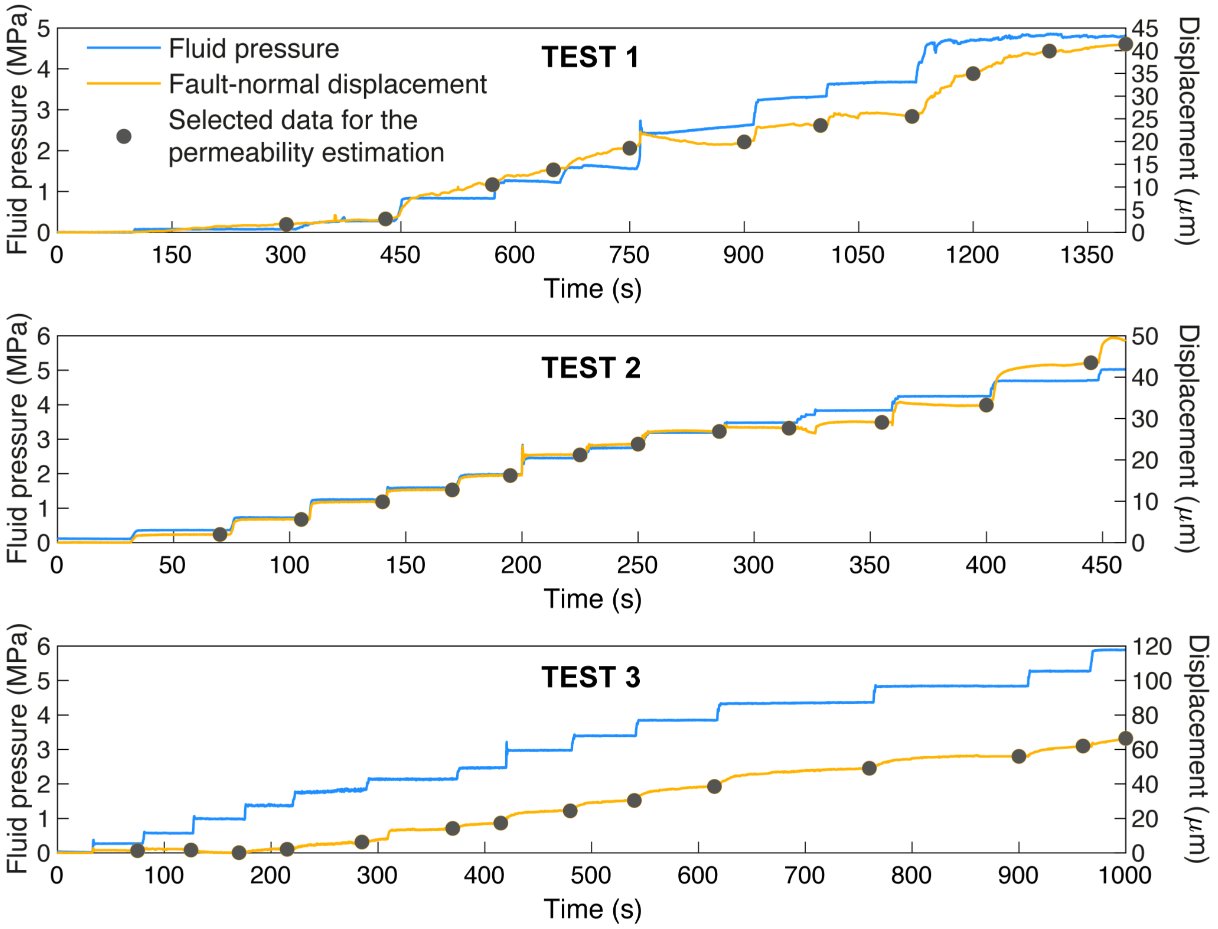

Supplementary Figure 4. Fluid pressure (blue) and fault-normal displacement (orange) measured during the three injection tests together with the selected data used for the estimation of permeability change with time (black dots).

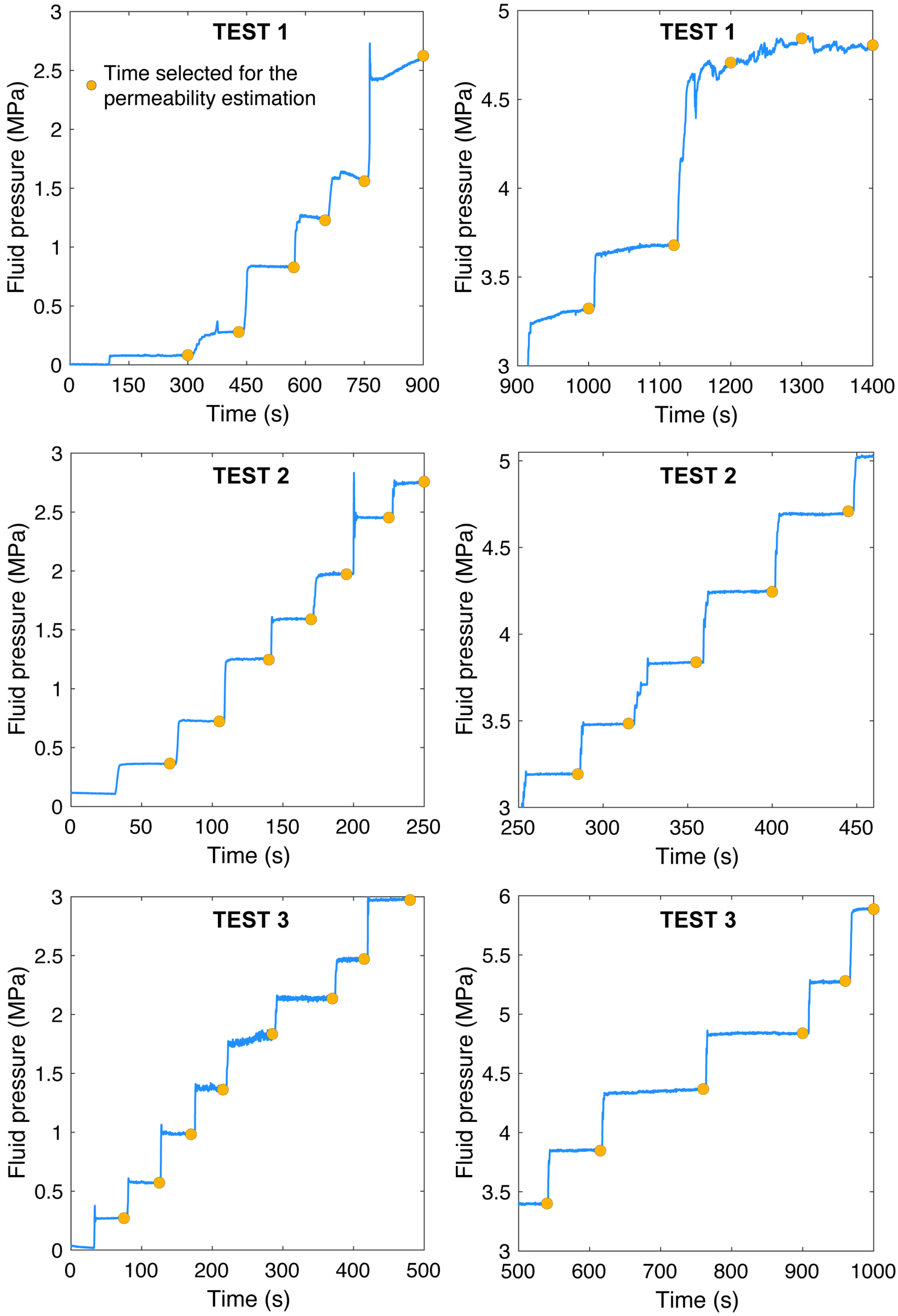

Supplementary Figure 5. Close-up view of the fluid pressure (blue) measured during the three injection tests together with the time selected for the estimation of permeability change (orange dots). (Left panels) Zoom over the pressure range from 0 to 3 MPa. (Right panels) Zoom over the pressure range from 3 MPa to the maximum injected pressure.

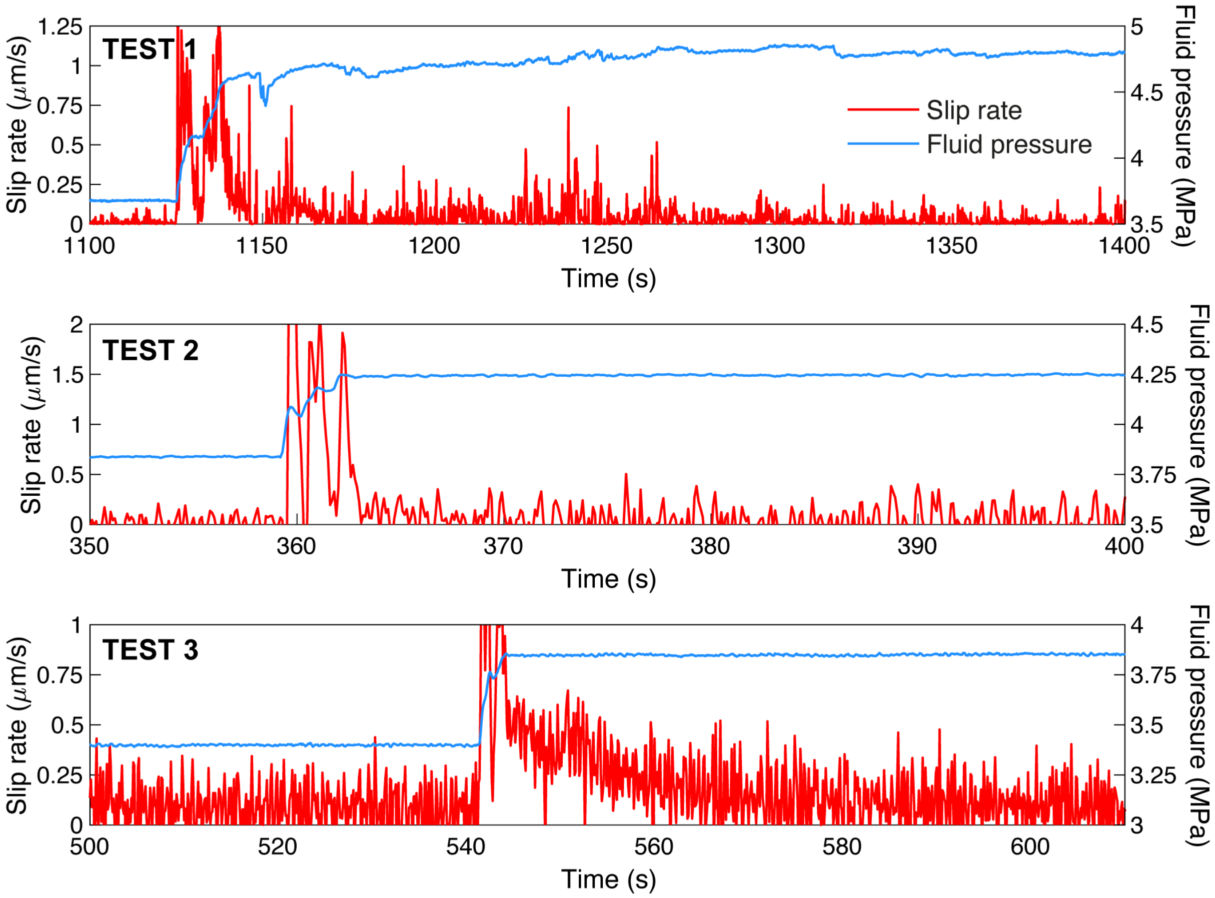

Supplementary Figure 6. Slip rate (red) and fluid pressure (blue) data as a function of time chosen for the three modelled sequences indicated in the yellow boxes in Figures S2 and S3.

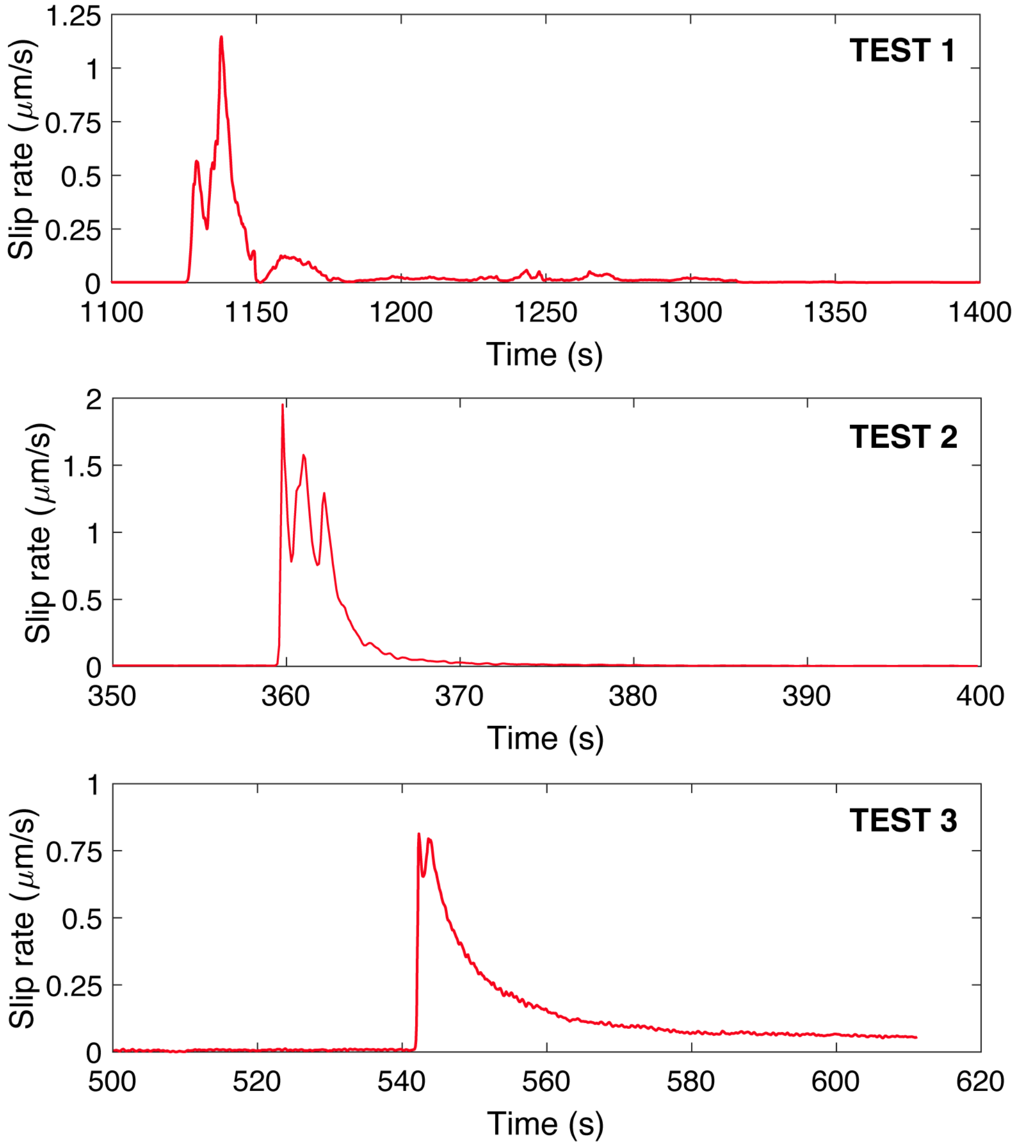

Supplementary Figure 7. Calculated slip rate as a function of time for the three modelled sequences indicated in the yellow boxes in Figures S2 and S3, and represented in close-up view in Figure S6.

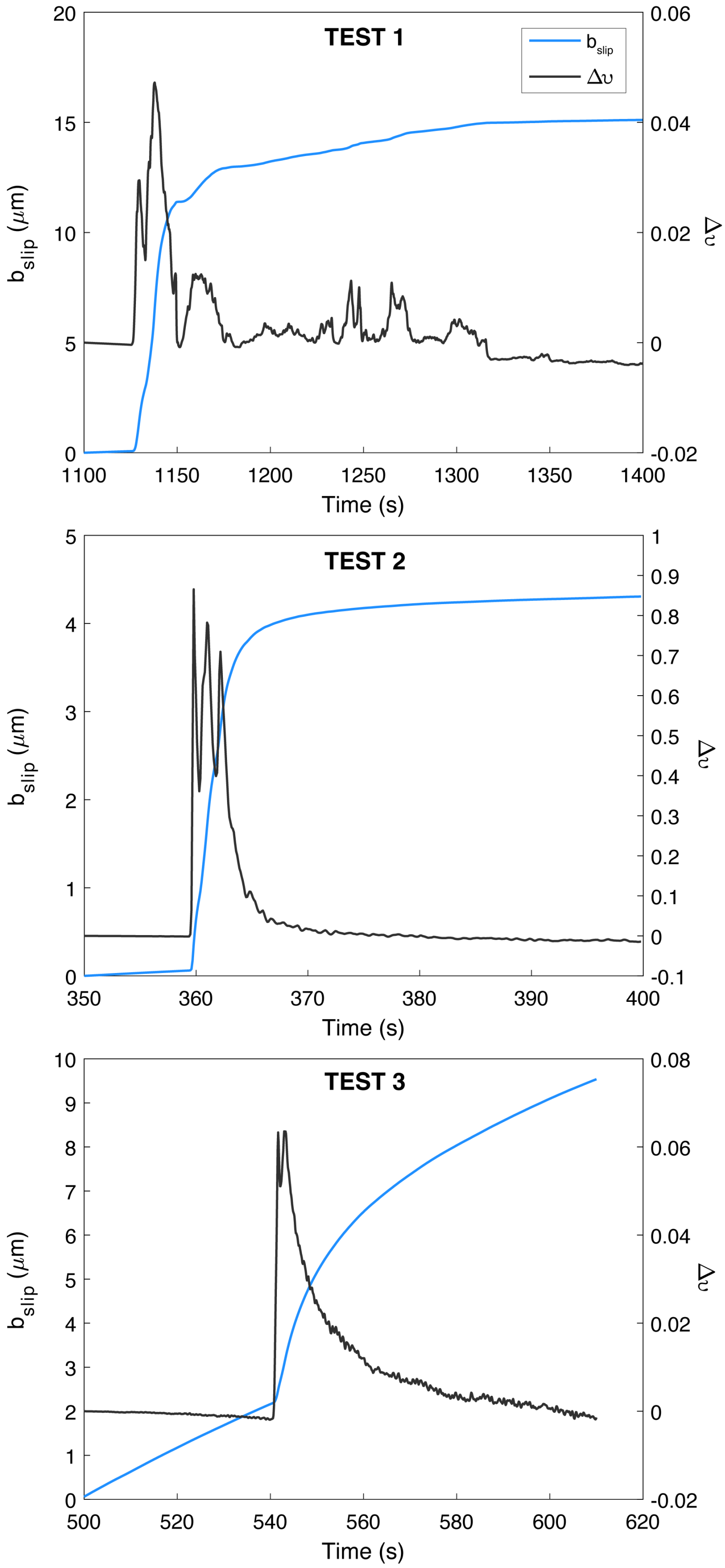

Supplementary Figure 8. Relative contribution of the slip-dependent hydraulic aperture (*"b_{slip}"* in blue) and the dilation parameter dependent on the slip velocity (*"Δυ"* in black) to the fault permeability change for the three injection tests.

Supplementary Tables.

Supplementary Table 1.

Fault frictional parameters inferred from the best-fit inversion of fault slip data during a step-increased pressure. The difference in fracture density and rock elasticity (Jeanne et al., 2012[20]) around the pressurized fault could explained the different value of K_s, while the difference in d_c could be due to different degree of roughness as observed in Jeanne et al., 2012[20].

Model parameters	Test 1	Test 2	Test 3
μ_0	0.5	0.5	0.5
a-b	-0.011	-0.028	0.01
d_c (μm)	5	48.75	50
v_0 (μm/s)	0.01	0.01	0.01
α	0.091	0.1	0.1
K_s (MPa/μm)	0.03	0.05	0.006
χ_r^2	0.594	0.018	0.061

Supplementary Table 2.

Fault hydraulic parameters inferred from the best-fit numerical solutions of fault slip and fault-normal displacement (opening) data during a step-increased pressure.

Model parameters	Test 1	Test 2	Test 3
β	3.325	2.8	1.8
γ	8.12×10^{-5}	8.12×10^{-5}	8.12×10^{-5}
b_0 (μm)	25.91	29.17	29.03
b_{max} (μm)	62.9	62.9	62.9
L (μm)	191.25	191.25	191.25
ψ	0.1	2.2	0.1
