# Supplementary material for: Transient evolution of permeability and friction in a slowly slipping fault activated by fluid pressurization
Source: Nat Commun. 2022 Jun 1;13:3039. doi: 10.1038/s41467-022-30798-3 (PMC9160226; doi:10.1038/s41467-022-30798-3)
Supplement: Supplementary file 3 — Description of Additional Supplementary Files [file 41467_2022_30798_MOESM3_ESM.pdf]

Descrtiption of Additional Supplementary Files

File name: Supplementary Dataset 1:
Description: Test_1_Data.mat contains the values of the time, fluid pressure, flowrate, fault aperture, fault slip, number of seismic events and distance of seismic events relative to the injection point measured in the Test 1. (Matlab file)

File name: Supplementary Dataset 2:
Description: Test_1_Permeability.mat contains the values of the fault permeability, fluid pressure and fault slip measured in the Test 1. (Matlab file)

File name: Supplementary Dataset 3:
Description: Test_2_Data.mat contains the values of the time, fluid pressure, flowrate, fault aperture, fault slip, number of seismic events and distance of seismic events relative to the injection point measured in the Test 2. (Matlab file)

File name: Supplementary Dataset 4:
Description: Test_2_Permeability.mat contains the values of the fault permeability, fluid pressure and fault slip measured in the Test 2. (Matlab file)

File name: Supplementary Dataset 5:
Description: Test_3_Data.mat contains the values of the time, fluid pressure, flowrate, fault aperture, fault slip, number of seismic events and distance of seismic events relative to the injection point measured in the Test 3. (Matlab file)

Description: File name: Supplementary Dataset 6:
Test_3_Permeability.mat contains the values of the fault permeability, fluid pressure and fault slip measured in the Test 3. (Matlab file)
